# DDRGK1 Enhances Osteosarcoma Chemoresistance via Inhibiting KEAP1‐Mediated NRF2 Ubiquitination

**DOI:** 10.1002/advs.202204438

**Published:** 2023-03-25

**Authors:** Xin Wang, Tangjun Zhou, Xiao Yang, Xiankun Cao, Gu Jin, Pu Zhang, Jiadong Guo, Kewei Rong, Baixing Li, Yibin Hu, Kexin Liu, Peixiang Ma, An Qin, Jie Zhao

**Affiliations:** ^1^ Shanghai Key Laboratory of Orthopaedic Implants Department of Orthopaedic Surgery Shanghai Ninth People's Hospital Shanghai Jiao Tong University School of Medicine 639 Zhaizaoju Road Shanghai 200011 P. R. China; ^2^ The Cancer Hospital of the University of Chinese Academy of Sciences (Zhejiang Cancer Hospital) Institute of Basic Medicine and Cancer (IBMC) Chinese Academy of Sciences Hangzhou 310022 P. R. China

**Keywords:** chemoresistance, DDRGK domain‐containing protein 1, doxorubicin, osteosarcoma, redox homeostasis

## Abstract

Chemoresistance is the main obstacle in osteosarcoma (OS) treatment; however, the underlying mechanism remains unclear. In this study, it is discovered that DDRGK domain‐containing protein 1 (DDRGK1) plays a fundamental role in chemoresistance induced in OS. Bioinformatic and tissue analyses indicate that higher expression of DDRGK1 correlates with advanced tumor stage and poor clinical prognosis of OS. Quantitative proteomic analyses suggest that DDRGK1 plays a critical role in mitochondrial oxidative phosphorylation. DDRGK1 knockout trigger the accumulation of reactive oxygen species (ROS) and attenuate the stability of nuclear factor erythroid‐2‐related factor 2 (NRF2), a major antioxidant response element. Furthermore, DDRGK1 inhibits ubiquitin‐proteasome‐mediated degradation of NRF2 via competitive binding to the Kelch‐like ECH‐associated protein 1 (KEAP1) protein, which recruits NRF2 to CULLIN(CUL3). DDRGK1 knockout attenuates NRF2 stability, contributing to ROS accumulation, which promotes apoptosis and enhanced chemosensitivity to doxorubicin (DOX) and etoposide in cancer cells. Indeed, DDRGK1 knockout significantly enhances osteosarcoma chemosensitivity to DOX in vivo. The combination of DDRGK1 knockdown and DOX treatment provides a promising new avenue for the effective treatment of OS.

## Graphical abstract

DDRGK domain‐containing protein 1 (DDRGK1) is considered a substrate of UFMylation. Here, the authors discover that independent of UFMylation, DDRGK1 can stabilize nuclear factor erythroid‐2‐related factor 2 (NRF2) by competing with Kelch‐like ECH‐associated protein 1 (KEAP1), thereafter inhibiting its ubiquitin‐mediated degradation. The loss of DDRGK1 leads to a decrease of NRF2, thus impairing the cellular antioxidant system. The accumulated ROS thereafter induces cell apoptosis and further enhances chemo‐sensitivity in cancer therapy.

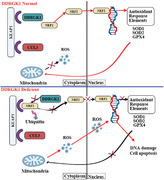

## 1 Introduction

Osteosarcoma (OS) is the most common primary bone tumor and is highly prevalent in children and adolescents.^[^ ^1^ ^]^ OS predominantly develops in the metaphysis of long bones, such as the femur, tibia, and humerus, where it aggresses local tissues and further develops into systemic metastases.^[^ ^2^ ^]^ A common therapy for patients with OS includes neoadjuvant treatment, which involves surgery and post‐surgery adjuvant therapy. Since the late 1970s, chemotherapies such as high‐dose methotrexate, etoposide, or ifosfamide combined with doxorubicin (DOX) or cisplatin have significantly elevated the 5‐year survival rate from less than 20% to >70%.^[^ ^3^ ^]^ However, due to genomic and individual heterogeneity, patients with similar clinical stages receiving the same type of treatment have different prognoses. In addition, ≈28% of patients with OS develop metastasis at first diagnosis or recurrence after chemotherapy and surgery, leading to amputation or death.^[^ ^4^ ^]^ Moreover, the overall survival rate has remained stagnant over the last two decades because of chemoresistance.^[^ ^5^ ^]^ Thus, there is an urgent clinical need for the development of drugs with different mechanisms of action to overcome chemoresistance in OS effectively.

Chemoresistance occurs via various molecular mechanisms, including hypoxia, endoplasmic reticulum (ER) stress, and reactive oxygen species (ROS). It is well documented that ROS is one of the vital agents responsible for chemoresistance.^[^ ^6^, ^7^ ^]^ ROS‐mediated mechanisms of resistance include the activation of redox‐sensitive transcription factors,^[^ ^8^ ^]^ switching from apoptosis to autophagy,^[^ ^9^ ^]^ and metabolic reprogramming.^[^ ^10^ ^]^ The relationship between oxidative stress and resistance to chemotherapy is reciprocal. Cells that are selected to survive mild or chronic ROS stimulation form chemoresistant phenotypes. In contrast, chemoresistant cancers are resistant to oxidative stress. In fact, compensatory elevated antioxidant activities are often observed in chemoresistant tumors, such as nuclear factor erythroid‐2‐related factor 2 (NRF2),^[^ ^11^ ^]^ adapted to higher ROS accumulation. Thereafter, perturbing the unstable redox balance, such as the elimination of the enzymes involved in antioxidant defense, would result in cell death^[^ ^8^ ^]^ and provide a promising strategy to overcome chemoresistance.

Emerging evidence indicates that ER stress and mitochondrial dysfunction are closely associated with ROS production.^[^ ^12^, ^13^ ^]^ Human DDRGK domain‐containing protein 1 (DDRGK1) is a highly conserved protein encoded by the short arm of chromosome 20 (20p13) and localizes in the peroxisomes, mitochondria, and mainly in the endoplasmic reticulum. It has been confirmed that DDRGK1 plays a vital role in endoplasmic reticulum homeostasis via regulation of IRE1a stability,^[^ ^12^ ^]^ thereby suggesting a potential role for DDRGK1 in the maintenance of redox homeostasis. To date, DDRGK1 is mostly considered a substrate of UFMylation, which conjugates the target protein with ubiquitin‐fold modifier 1 (UFM1).^[^ ^14^ ^]^ Although it has recently been shown to play a role in UFMylation‐mediated regulation of erythroid development,^[^ ^15^ ^]^ spondyloepimetaphyseal dysplasia,^[^ ^16^ ^]^ and plasma cell development,^[^ ^17^ ^]^ the mechanism by which DDRGK1 regulates UFMylation and its other functions remain largely unknown. In fact, the identification of DDRGK1‐interacting proteins using label‐free quantitative proteomics showed that DDRGK1 is involved in regulating protein folding, stability, and trafficking.^[^ ^18^ ^]^ This result suggested that DDRGK1 might play a more important role than previously discovered. Indeed, deletion of DDRGK1 results in embryonic lethality in vivo, suggesting its indispensable role in cell growth^[^ ^15^ ^]^ and most studies consider DDRGK1 to be a cytoprotective protein.^[^ ^12^, ^16^ ^]^ In terms of tumor biology, despite its high expression in tumors such as squamous cell carcinomas and lung adenocarcinomas, there is little evidence in support of it being considered an oncogene.^[^ ^19^ ^]^ In fact, the function of DDRGK1 in tumor development remains controversial. For example, DDRGK1 interacts with I*κ*B*α* and regulates its stability, and its deletion inhibits tumor cell proliferation.^[^ ^20^ ^]^ Furthermore, by interacting with activating signal cointegrator 1 (ASC1), DDRGK1 regulates ASC1 UFMylation and promotes ER‐positive breast cancer development.^[^ ^21^ ^]^ However, DDRGK1 has also been shown to maintain p53 stability and impair the growth of colon cancer via cooperation with UFM1 and covalent modification of p53.^[^ ^22^ ^]^ Without further research, it is difficult to fully characterize the function of DDRGK1 in tumors.

Since ROS are usually upregulated in tumor cells, they rely heavily on the antioxidant system to effectively keep ROS below lethal levels and are more vulnerable to any insults that disrupt ROS homeostasis.^[^ ^23^, ^24^ ^]^ Here, we report a novel role of DDRGK1 in regulating key ROS proteins by competing with NRF2 to bind to Kelch‐like ECH‐associated protein 1 (KEAP1), blocking the initiation of ubiquitin‐proteasome‐mediated degradation by CUL3 (an E3 ligase of NRF2), thus broadly regulating the antioxidant system. We showed that deleting DDRKG1 induces cellular ROS accumulation, leading to apoptosis and attenuation of DOX chemoresistance, further suppressing tumor formation in vivo. Taken together, these results reveal a crucial role of DDRGK1 in the KEAP1/NRF2/ROS pathway, which contributes to osteosarcoma development and chemoresistance.

## 2 Results

### 2.1 High‐Level Expression of DDRGK1 Correlates with Poor Clinical Prognosis

**Figure 1:**
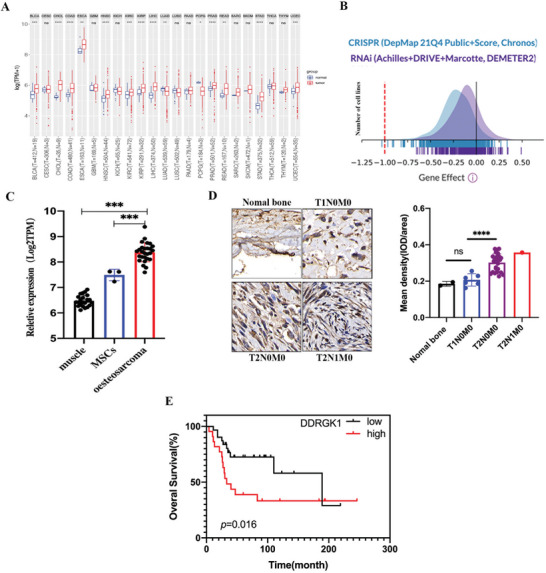
DDRGK1 acts as a cancer‐promoting gene. A) Pan‐cancer analysis of expression of DDRGK1 in different tumors compared with normal tissues using TCGA database. B) Gene dependency analysis of DDRGK1 using Depmap database. Negative values represent gene knockdown would inhibit cell growth. C) mRNA levels of DDRGK1 in muscle, mesenchymal stem cells, and osteosarcoma tissues, data were extracted from GSE18043, GSE38718, and GSE14827 respectively. D) Levels of DDRGK1 in bone and osteosarcoma with different clinical stages by immunohistochemistry staining. The OS tissue array was purchased from Biomax (CAT:OS804d) including 40 cases. E) Kaplan–Meier survival analysis of DDRGK1 for osteosarcoma according to the GSE21257 dataset. (***p < 0.001, ****p < 0.0001).

Pan‐cancer analysis was performed based on The Cancer Genome Atlas (TCGA) Pan Cancer database. Of the 23 selected cancers, 14 showed high DDRGK1 expression in tumor tissue when compared to adjacent normal tissue, while only one tumor (pheochromocytoma and paraganglioma) showed lower expression of DDRGK1 compared to normal tissue (**Figure** **1**A), suggesting that DDRGK1 may act as a cancer‐promoting gene in most tumors. Subsequently, we analyzed data from the DepMap database, which includes 1055 cell lines from multiple systems. The score evaluates the effect of knocking out or knocking down a gene while normalizing expression against the distribution of pan‐essential and non‐essential genes.^[^ ^25^, ^26^ ^]^ A negative score indicated that the gene knockdown inhibited cell growth. We found that most cell lines were strongly dependent on DDRGK1 because the score was mostly distributed in the negative region (Figure 1B). To further distinguish the significant difference between normal and OS samples, we integrated three OS Gene Expression Omnibus (GEO) datasets (GSE18043, GSE38718, and GSE14827) and found that expression of DDRGK1 in OS tissues was higher than that in mesenchymal stem cells and muscle tissue (Figure 1C). In addition, DDRGK1 expression correlated with the Tumor Node Metastasis (TNM) stage (Figure 1D). Furthermore, Kaplan–Meier analyses of overall survival were performed based on a GEO DataSet (GSE21257) that contained data for 53 patients with OS. We found that high levels of DDRGK1 expression correlated with a shorter lifespan (Figure 1E). Taken together, these results indicate the oncogenic role of DDRGK1 in OS and suggest that it may act as a poor prognostic marker.

### 2.2 DDRGK1 Promotes Osteosarcoma Cell Growth

**Figure 2:**
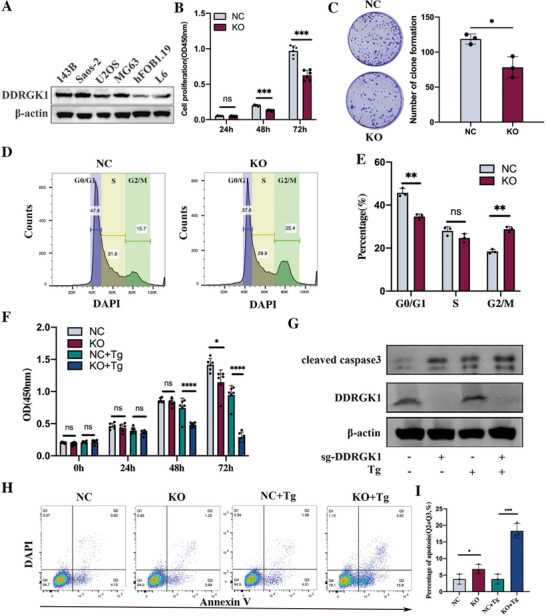
DDRGK1 promotes cell proliferation and inhibits apoptosis. A) Expression of DDRGK1 in various cell lines including osteosarcoma cell lines 143B, Saos‐2, U2OS and MG63, osteoblast cell line hFOB1.19, and muscle cell line L6 were detected by western blot. B) Effects of DDRGK1 on cell proliferation in osteosarcoma cells. Wild‐type 143B and DDRGK1‐knockout 143B cells were cultured at different times and cell viability was detected by CCK‐8 assay. C) Effect of DDRGK1 on cell clone formation. Wild‐type 143B and DDRGK1‐knockout 143B cells with 800 cells per well were seeded in a plate and cultured for 7 days, followed by crystal violet staining. D) Effects of DDRGK1 on cell cycle. Wild‐type 143B and DDRGK1‐knockout 143B cells were seeded in plate and hungered without serum for 24 h, then cultured for another 24 h with serum and collected for Flow cemetery analysis. E) Statistical analysis of cell cycle. F) Cell toxicity induced by thapsigargin (Tg) with or without DDRGK1. Wild‐type 143B and DDRGK1‐knockout 143B cells were subjected to Tg (10 nM) for the indicated time, and cell viability was detected by CCK‐8 assay. G) Influence of apoptosis after DDRGK1 knockout. Cleaved caspase 3 represents cell apoptosis was detected by western blot in cell lysates from Wild‐type 143B cells and DDRGK1‐knockout 143B with or without subjected to Tg (10 nM, 24 h). H) Flow cemetery to detect apoptosis rate by Annexin V/DAPI staining after being treated with Tg (10 nM) for 24 h. I) Statistical analysis of cell apoptosis. (three samples for each statistical analysis, *p < 0.05; **p < 0.01; ***p < 0.001).

Motivated by the bioinformatic findings, we evaluated the expression pattern of DDRGK1 in various OS cell lines in comparison to the osteoblast cell line Hfob1.19, and the skeletal muscle cell line L6. We found that the protein expression of DDRGK1 in OS cells was higher than that in both osteoblasts and muscle cells (**Figure** **2**A). 143B cells with higher malignancy expressed more DDRGK1 than those with lower malignancy (Saos‐2, U2OS, and MG63) (Figure S1, Supporting Information). Based on the expression levels of DDRGK1, 143B, and Saos‐2 cells were selected for further study.

DDRGK1 knockout 143B and Saos‐2 cell lines were constructed using CRISPR‐Cas9. CCK8 assay demonstrated that DDRGK1 knockout significantly inhibited the proliferation of both 143B and Saos‐2 cells in a time‐dependent manner (Figure 2B, Figure S2A, Supporting Information). Moreover, without DDRGK1 expression, the number of colonies significantly decreased (Figure 2C and Figure S2B, Supporting Information). Next, we investigated its effects on cell cycle progression. G2M arrest occurred in both 143B and Saos‐2 cells, indicating that they may have entered mitosis before DNA repair, leading to cell death (Figure 2D,E and Figure S2C,D, Supporting Information). The PI3K‐AKT pathway is important for cell proliferation.^[^ ^27^ ^]^ As expected, the levels of phosphorylated AKT and PI3K were reduced when DDRGK1 was knocked out, suggesting impairment of the PI3K‐AKT pathway (Figure S2E, Supporting Information). These results demonstrated that DDRGK1 knockout significantly impaired cell proliferation.

DDRGK1 has been reported to induce ER stress and enhance ER stress‐induced apoptosis in cancer cells by regulating IRE1a‐XBP1 signaling.^[^ ^12^, ^17^ ^]^ To further confirm the role of DDRGK1 in osteosarcoma cell apoptosis, we used the ER stress‐related apoptosis inducer thapsigargin (Tg).^[^ ^28^ ^]^ We observed a Tg‐induced time‐dependent inhibition of proliferation. Furthermore, DDRGK1 knockout rendered cells more sensitive to Tg, especially after 72 h, when nearly all the cells were dead (Figure 2F). As determined via microscope, following treatment with Tg (20 nM) for 24 h, adherent cells, tending to undergo apoptosis, started to detach from the plate and were suspended in a culture medium. More dead cells were observed in the DDRGK1 knockout cells than in the normal cells (Figure S3, Supporting Information). As cleaved caspase 3 is a marker of cell apoptosis, we compared its level in DDRGK1 knockout cells and wild‐type cells. In line with the flow cytometry results, the level of cleaved caspase 3 also increased in the DDRGK1 knockout cells and further increased upon treatment with Tg compared to normal cells (Figure 2G). Flow cytometry was used to quantify apoptosis following Annexin V and 4,6‐diamidino‐2‐phenylindole (DAPI) staining. Without Tg, the apoptosis ratio was elevated when DDRGK1 was knocked out, and it increased in the presence of Tg (Figure 2H,I). Taken together, these results suggest that DDRGK1 plays a vital role in the regulation of OS cell growth.

### 2.3 DDRGK1 Modulates Mitochondrial Metabolism and Cell Antioxidant Capacity

**Figure 3:**
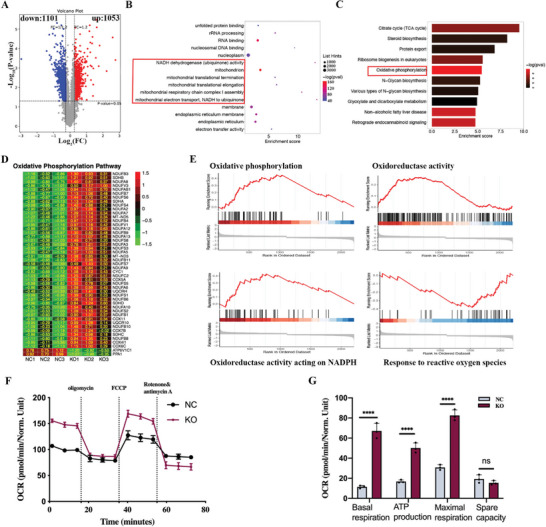
Quantitative Proteomics analyses reveal the function of DDRGK1. A) Volcano plot for differentially expressed genes. Whole‐cell protein extracts of DDRGK1 knockout 143B cells and control cells were harvested, labeled with TMT then followed by mass spectrometry. A total of 2154 differentially expressed proteins were identified with 1053 proteins upregulated and 1101 downregulated. B) Gene Ontology enrichment analysis for differentially expressed genes. C) KEGG analysis for differentially expressed genes. D) Heatmap for expression of oxidative phosphorylation‐related proteins in DDRGK1 knockout cells and the control cells. Expression values were normalized by the “pheatmap” R package. E) Gene Set Enrichment Analysis (GSEA) in DDRGK1 knockout cells and the control cells. F) Mitochondrial metabolism analysis. Cells were seeded in an XFmicroplate at an optimal density and cultured for 24 h. The cellular oxygen consumption rate (OCR) was measured using a Seahorse XF Analyzer. G) Mitochondrial respiratory capacity analysis according to the OCR. (three samples for each statistical analysis, *p < 0.05; ****p < 0.0001).

To reveal the molecular mechanisms underlying DDRGK1‐mediated tumor growth, tandem mass tag (TMT)‐based quantitative proteomics was performed using 143B cells following DDRGK1 knockout. A total of 2154 differentially expressed proteins were identified as having a >1.2‐fold change in relative abundance (*p* < 0.05), of which 1053 proteins were upregulated and 1101 were downregulated when compared to control cells (**Figure** **3**A). Next, we investigated the top 15 significantly enriched GO (Gene Ontology) terms. The most enriched pathway was related to mitochondrial activity, including NADH dehydrogenase (ubiquinone) activity, mitochondrial translational termination, mitochondrial translational elongation, mitochondrial respiratory chain complex I assembly, mitochondrial electron transport, and NADH to ubiquinone, indicating a significant influence of DDRGK1 on the mitochondrial metabolic activity (Figure 3B). KEGG pathway analysis also revealed that mitochondrial oxidative phosphorylation was enriched and that proteins relevant to oxidative phosphorylation, especially components of ECT (electron transport chain) complex I, such as NDUFA, NDUFB, NDUFAB, and NDUFV, were upregulated in DDRGK1 knockout cells (Figure 3C,D), indicating an increase in mitochondrial metabolism after DDRGK1 knockout. Gene Set Enrichment Analysis (GSEA) identified DDRGK1 as being broadly associated with oxidative phosphorylation, oxidoreductase activity, oxidation‐reductase activity acting on NADPH, and response to reactive oxygen species. More importantly, the response to the ROS pathway was found to be enriched in control cells, while the other three were enriched in DDRGK‐knockout cells (Figure 3E). Taken together, these results indicate that DDRGK1 knockout results in high oxidative activity and impaired antioxidant capacity in cells.

To further explore the mechanism, metabolic analysis of mitochondria was performed using Seahorse technology. The oxygen consumption rate (OCR) of mitochondria was significantly enhanced after DDRGK1 knockout (Figure 3F). Initially, basal respiration was elevated at rest. After inhibiting basal respiration with oligomycin, adenosine triphosphate (ATP) production in knockout cells increased significantly. Furthermore, maximal respiration was enhanced after aerobic respiration was reactivated by FCCP in DDRGK1 knockout cells (Figure 3G). These results indicated that DDRGK1 plays a critical role in the regulation of mitochondrial respiration and energy metabolism.

### 2.4 DDRGK1 Regulates ROS Production by Adjusting Stability of NRF2

**Figure 4:**
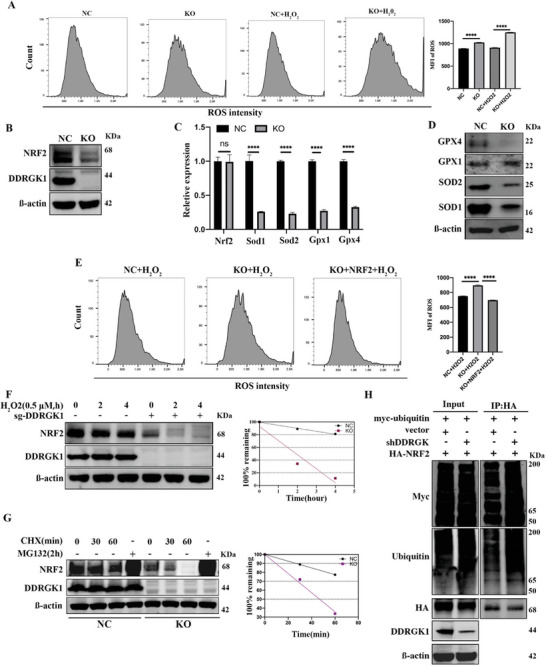
DDRGK1 regulates ROS production by regulating NRF2 stability. A) Sensitivity of DDRGK1‐knockout cells to H_2_O_2_. The controlled and DDRGK1‐knockout cells were subjected to H_2_O_2_ for 20 min, then loaded with a DHE probe for 30 min and detected by flow cemetery. B) Regulation of NRF2 by DDRGK1. The protein levels of NRF2 in controlled cells and DDRGK1‐knockout 143B cells were detected by western blot. C) Regulation of NRF2 downstream mRNA levels by DDRGK1. The mRNA levels of NRF2 and its downstream genes in controlled cells and DDRGK1‐knockout 143B cells were detected by qPCR. D) Regulation of downstream proteins of NRF2 by DDRGK1. The expression of SOD1, SOD2, GPX1, and GPX4 in controlled cells and DDRGK1‐knockout 143B cells were detected by western blot. E) NRF2 overexpression rescues the ROS accumulation induced by H_2_O_2_. NRF2‐HA plasmid was transfected into DDRGK1‐knockout 143B cells, cells were subject to H_2_O_2_ and ROS levels were detected by flow cemetery. F) Kinematic decrease of NRF2 under H_2_O_2_ stimulation. The cells were treated with H_2_O_2_ for the indicated times and NRF2 expression was detected by western blot. G) Stability of NRF2 after DDRGK1 knockout. The DDRGK1 knockout and controlled 143B cells were treated with cycloheximide (50 µg mL^−1^) and MG132 (10 µM) for indicated times. NRF2 levels were detected by western blot. H) Ubiquitination of NRF2. NRF2‐HA, ubiquitin‐Myc plasmids were transfected into HEK293T cells with or without co‐transfected with sh‐DDRGK1 plasmid. Ubiquitination level was detected by immunoprecipitation with HA antibody followed by western blot with anti‐ubiquitin antibody. (MFI, mean fluorescence intensity; Three samples for each statistical analysis, ****p < 0.0001).

Electrons released from the mitochondrial electron transport chain play an important role in ROS production.^[^ ^29^ ^]^ Elevated oxidative phosphorylation and impaired antioxidant capacity theoretically result in the release of electrons and ROS accumulation. To determine whether the metabolic changes caused by DDRGK1 were accompanied by ROS accumulation, we used flow cytometry to measure intracellular H_2_O_2_‐induced ROS levels under physiological and pathological conditions. As expected, ROS levels were significantly elevated under both conditions, but DDRGK1 knockout cells seemed to be more sensitive to pathological conditions than the control group (**Figure** **4**A). NRF2 is one of the major regulators of the endogenous antioxidant system and serves as a transcription factor that regulates the expression of a broad range of antioxidant response element (ARE) genes involved in antioxidant responses. We next investigated the expression pattern of NRF2 and found that it was significantly decreased in DDRGK1‐knockout 143B and Saos‐2 cells (Figure 4B and Figure S4A, Supporting Information), similar to that in DDRGK1‐silenced HEK293T cells (Figure S4B, Supporting Information). In contrast, inhibition or activation of NRF2 did not affect DDRGK1 expression, indicating that DDRGK1 acts upstream of NRF2 (Figure S4C,D, Supporting Information). The mRNA expression of ARE genes, including SOD1, SOD2, GPX1, and GPX4, decreased (Figure 4C). The protein expression levels of these markers, except GPX1, were also decreased (Figure 4D and Figure S5A, Supporting Information). As the current DDRGK1 knockout system represents a chronic impact on NRF2, it is unknown if any differences exist when DDRGK1 is acutely depleted. Therefore, we used DDRGK1 siRNA and a doxycycline‐inducible system to acutely downregulate DDRGK1 expression. Similar to the results in DDRGK1 knockout cells, downregulating DDRGK1 via both methods had no significant effect on the mRNA level of NRF2 but significantly decreased the mRNA expression of ARE genes regulated by NRF2 (Figure S5B,C, Supporting Information). In addition, we analyzed the above‐mentioned quantitative proteomics results to characterize the expression pattern of additional ARE proteins, further confirming that most ARE proteins were indeed downregulated in DDRGK1 knockout cells (Figure S5D, Supporting Information). Moreover, overexpression of NRF2 successfully rescued ROS accumulation resulting from DDRGK1 knockout (Figure S6, Supporting Information and Figure 4E). These data suggest that DDRGK1 knockout leads to decreased NRF2 expression, which further regulates the expression of its downstream ARE gene.

Next, we found that NRF2 showed a time‐dependent decrease after treatment with H_2_O_2_ which occurred more quickly in DDRGK1 knockout cells (Figure 4F). As NRF2 mRNA levels did not decrease (Figure 4C), we hypothesized that the regulation of NRF2 by DDRGK1 may involve a post‐transcriptional mechanism. Thus, we examined the effect of DDRGK1 on NRF2 stability in cells treated with cycloheximide, which disrupts protein translocation, and found that NRF2 stability decreased in DDRGK1 knockout cells (Figure 4G). Treatment of cells with the proteasome inhibitor MG132 blocked the DDRGK1‐mediated reduction in NRF2 protein levels (Figure 4G). In addition, DDRGK1 knockout increased the ubiquitination of NRF2 (Figure 4G). Together, these results indicated that DDRGK1 regulates NRF2 stability, thereby regulating the cellular response to ROS accumulation.

### 2.5 DDRGK1 Regulate NRF2 via UFMylation Independent Pathway

**Figure 5:**
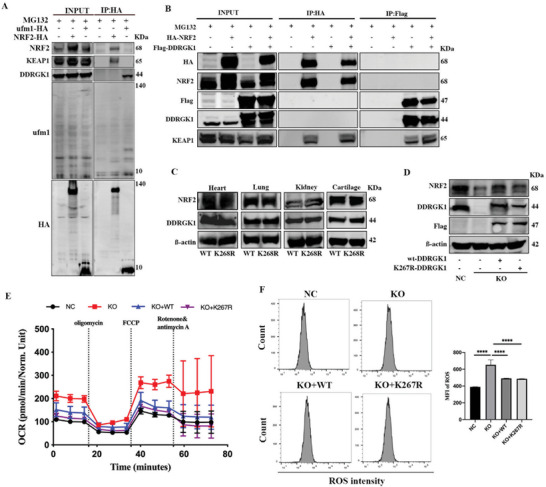
DDRGK1 regulates NRF2 in a UFMylation‐independent pathway. A) Interaction between NRF2 and UFM1. The NRF2‐HA and ufm1‐HA plasmids were transfected into HEK293T cells. The interaction between NRF2 and ufm1 was detected by co‐immunoprecipitation with HA antibody followed by western blot with NRF2 and ufm1 antibody. B) Interaction between NRF2 and DDRGK1. The NRF2‐HA and DDRGK1‐Flag plasmids were transfected into HEK293T cells. The interaction between NRF2 and DDRGK1 was detected by co‐immunoprecipitation using anti‐HA immunomagnetic beads and anti‐Flag immunomagnetic beads. C) NRF2 levels of heart, lung, kidney, and cartilage in WT and K268R mouse detected by western blot. D) Re‐express of WT‐DDRGK1 and K267R‐DDRGK1 in DDRGK1 deficient cells rescued level of NRF2. E) Mitochondrial metabolism analysis. Cells were seeded in an XF microplate at an optimal density and cultured for 24 h. The cellular oxygen consumption rate (OCR) was measured using a Seahorse XF Analyzer. F) ROS level measurement. The controlled, DDRGK1‐knockout cells, and WT‐DDRGK1/K267R‐DDRGK1 re‐expressed cells were subjected to H_2_O_2_, then loaded with a DHE probe for 30 min and detected by flow cemetery. (MFI, mean fluorescence intensity; Three samples for each statistical analysis ****p < 0.0001).

As DDRGK1 was identified as a substrate of UFMylation,^[^ ^14^ ^]^ we investigated whether the regulation of NRF2 by DDRGK1 is involved in UFMylation. During UFMylation, in addition to direct interaction with UFM1, DDRGK1 is required for other UFMylation substrates, including ASC1, RPL26, and RPN1.^[^ ^30^, ^31^ ^]^ Therefore, if DDRGK1 regulates the UFMylation of a target protein, two scenarios are necessary: 1) the target protein should interact with Ufm1 and 2) the target protein interacts with DDRGK1. Therefore, we tested these two scenarios. First, HA‐tagged NRF2 and Ufm1 plasmids were co‐transfected into HEK293T cells to investigate whether NRF2 interacted with Ufm1. No interaction was detected between NRF2 and Ufm1 (**Figure** **5**A). Second, no interaction was found between DDRGK1 and NRF2, regardless of the use of FLAG‐tagged DDRGK1 to co‐immunoprecipitate NRF2 or HA‐tagged NRF2 to co‐immunoprecipitate DDRGK1 (Figure 5B). Since Lys‐267 of DDRGK1 is reported to be the main lysine residue for Ufm1 conjugation in the UFMylation process,^[^ ^14^ ^]^ we constructed a K268R C57BL/6NGpt mouse, in which the lysine residue at position 268 of DDRGK1 was substituted with an arginine residue (mouse K268R corresponds to human K267R), and the NRF2 levels in the lung, heart, kidney, and cartilage in K268R mice were compared with those in WT mice. We found that K268R did not influence NRF2 expression in these organs (Figure 5C), indicating that NRF2 was not a target of UFMylation. To further confirm that the decrease in NRF2 was DDRGK1‐dependent and UFMylation‐independent, we performed complementation experiments in which we expressed WT‐DDRGK1 or K267R‐DDRGK1 in DDRGK1 deficient cells. Both WT‐DDRGK1 and K267R‐DDRGK1 rescued the levels of NRF2 (Figure 5D), mitochondrial OCR (Figure 5E), and ROS (Figure 5F) in DDRGK1 deficient cells. Therefore, our data established that DDRGK1 regulates NRF2 via a UFMylation‐independent pathway.

### 2.6 DDRGK1 Competes with NRF2 for KEAP1 Binding Thereby Inhibiting NRF2 Degradation

**Figure 6:**
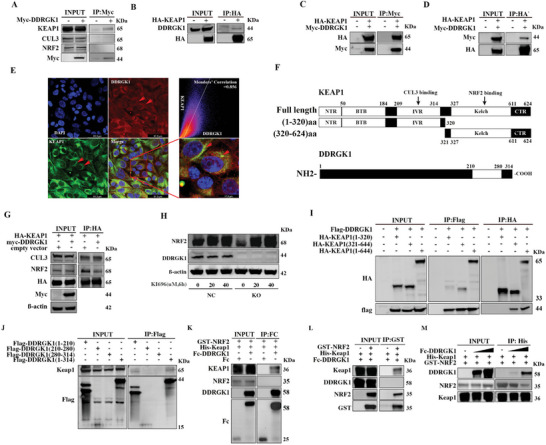
DDRGK1 interacts with KEAP1. A) Interactions between DDRKG1 and NRF2, KEAP1, or CUL3. HEK293T cells were transfected with Myc‐DDRGK1 plasmid followed by co‐immunoprecipitation with Myc antibody. B) Interactions between DDRKG1 and KEAP1. HEK293T cells were transfected with HA‐KEAP1 plasmid then co‐immunoprecipitation with HA antibody followed by western blot with DDRGK1 antibody. C) Interactions between DDRKG1 and KEAP1. HEK293T cells were co‐transfected with HA‐KEAP1 and Myc‐DDRGK1 plasmids followed by co‐immunoprecipitation with Myc antibody and western blot with HA antibody. D) Interactions between DDRKG1 and KEAP1. HEK293T cells were co‐transfected with HA‐KEAP1 and Myc‐DDRGK1 plasmids then co‐immunoprecipitation with HA antibody followed by western blot with Myc antibody. E) Co‐localization of DDRGK1 and KEAP1 by immunofluorescence staining and observed under confocal microscopy. F) Domains and binding sites in Keap1 protein and truncating strategy for constructing different lengths of KEAP1 plasmids and DDRGK1 plasmids. G) DDRGK1 affects affinity between KEAP1 and NRF2, rather than with CUL3. HEK293T cells were transfected with HA‐KEAP1 and with or without co‐transfected with Myc‐DDRGK1 plasmid, then co‐immunoprecipitation with HA antibody. H) KI696 rescued NRF2 levels in DDRGK1 knockout cells. I) Identifying the binding site in Keap1 to interact with DDRGK1. Different lengths of KEAP1 plasmids and flag‐DDRGK1 plasmid were transfected into HEK293T cells respectively, and then co‐immunoprecipitation with HA antibody followed by western blot with flag antibody. J) Identifying the binding site in DDRGK1 to interact with KEAP1. Different lengths of DDRGK1 plasmids were transfected into HEK293T cells respectively, and then co‐immunoprecipitation with Flag antibody followed by western blot with KEAP1 antibody. K) Fc pulldown assay. 10 µg purified Fc, Fc‐DDRGK1 were mixed with KEAP1 and NRF2 protein, incubated with protein A/G beads, then detected by Co‐IP procedures. L) GST pulldown assay. 10 µg GST‐NRF2 protein was mixed with KEAP1 and DDRGK1 protein, incubated with protein GST beads, then detected by Co‐IP procedures. M) His pulldown experiment. 10 µg His‐KEAP1 protein was mixed with NRF2 and 5 or 10 µg DDRGK1 protein, incubated with His beads, then detected by Co‐IP procedures.

As previously reported, NRF2 degradation is regulated by a KEAP1‐dependent pathway, and KEAP1 serves as an adaptor protein for CULLIN 3 (CUL3), an E3 ubiquitin ligase.^[^ ^32^ ^]^ Under unstressed conditions, KEAP1 recruits CUL3 to form a complex with NRF2, resulting in ubiquitination and rapid degradation of NRF2 via the proteasome, thus maintaining low NRF2 levels and activity.^[^ ^33^ ^]^ Once KEAP1 senses redox‐disruptive stimuli, its thiols can be directly modified, leading to inactivation and NRF2 degradation.^[^ ^34^ ^]^ To determine whether DDRGK1 is involved in this pathway, we measured KEAP1 and CUL3 expression levels in DDRGK1 knockout cells and found that both mRNA and protein expression levels were unchanged compared to the control (Figure S8A,B, Supporting Information). To test whether DDRGK1 interacts with NRF2, KEAP1, or CUL3, we performed a reciprocal co‐immunoprecipitation (co‐IP) assay in HEK293T cells. When using Myc‐tagged DDRGK1 to co‐immunoprecipitate other endogenous proteins, no direct interaction with NRF2 or CUL3 was observed (**Figure** **6**A). Importantly, we found a direct interaction between DDRGK1 and KEAP1 (Figure 6A), and the same interaction was observed when HA‐tagged KEAP1 was used to co‐IP endogenous DDRGK1 (Figure 6B). To further confirm these results, we tested this interaction exogenously by co‐transfecting HA‐KEAP1 and Myc‐DDRGK1 plasmids into 293T cells. We detected the same reciprocal interaction in both Myc‐DDRGK1 and HA‐KEAP1 immunoprecipitates (Figure 6C,D). Confocal microscopy and immunofluorescence staining revealed co‐localization of DDRGK1 and KEAP1 in the cytoplasm, especially around the nucleus (Manders’ co‐localization coefficient = 0.856; Figure 6E), which is consistent with the finding that KEAP1 localizes in the perinuclear cytoplasm with loose attachment to the actin cytoskeleton.^[^ ^34^ ^]^

Previous studies reported three KEAP1 domains, including a broad‐complex, tramtrack, and bric‐à‐brac (BTB) domain, an intervening region (IVR) responsible for interacting with CUL3, and a double‐glycine repeat (DGR, Kelch) domain that allows interaction with NRF2 (Figure 6F).^[^ ^35^ ^]^ We constructed HEK293T cells stably expressing KEAP1 and DDRGK1 to investigate their relationship. We found that the interaction between KEAP1 and NRF2 was reduced by DDRGK1 overexpression, whereas the interaction between KEAP1 and CUL3 was unchanged compared to the control (Figure 6G). These data suggest that DDRGK1 may contribute to NRF2 degradation by competing with NRF2 for binding to KEAP1 via its Kelch domain, thereby stopping CUL3‐initiated degradation. To confirm this speculation, KI696, a small molecule that precludes the KEAP1‐NRF2 interaction, was used. KI696 rescued the protein level of NRF2 in DDRGK1 deficient cells (Figure 6H), which demonstrated that DDRGK1 may interfere with the interaction between KEAP1 and NRF2. Furthermore, we constructed two plasmids expressing amino acids 1–320 and 321–624 of KEAP1 (Figure 6F). The co‐IP results showed the binding of DDRGK1 with amino acids 321–624 of KEAP1, which is the segment containing the Kelch domain, rather than with amino acids 1–320 (Figure 6I). Furthermore, to map the domain in DDRGK1 that interacts with KEAP1, we constructed three plasmids expressing amino acids 1–210, 210–280, and 280–314 of DDRGK1. Co‐IP results showed the binding of KEAP1 to amino acids 1–210 of DDRGK1 (Figure 6J).

To further prove the direct interaction between DDRGK1, NRF2, and KEAP1, we purified Fc‐tagged DDRGK1 (full length), Fc protein, His‐tagged KEAP1 (C‐terminal amino acids 312–624, containing the Kelch domain of KEAP1), and GST‐tagged NRF2 (N‐terminal amino acids 1–84, containing domains interacting with KEAP1) proteins (Figure S9, Supporting Information) and performed pulldown experiments. Similar to the results obtained using mammalian cell lysis, no direct interaction between DDRGK1 and NRF2 was observed (Figure 6K,L). DDRGK1 directly interacted with the Kelch domain of KEAP1 (Figure 6K) and competed with NRF2 for this binding domain (Figure 6M).

Similarly, we investigated whether UFMylation is responsible for the interaction between DDRGK1 and KEAP1. Immunoprecipitation experiments revealed that the K267R mutant did not disturb conjugation with HA‐KEAP1 (Figure S10, Supporting Information). Taken together, these results suggest that DDRGK1 binds competitively with KEAP1, independent of UFMylation, thus inhibiting NRF2 degradation.

### 2.7 DDRGK1 Enhances Doxorubicin Chemoresistance by Regulating ROS Accumulation

**Figure 7:**
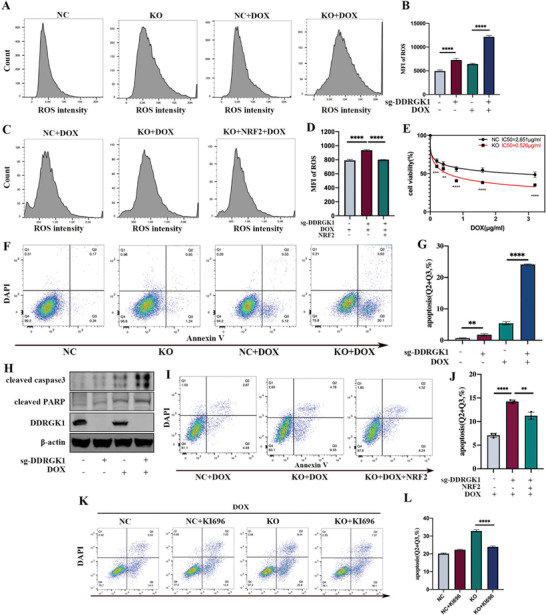
DDRGK1 knockout enhances chemosensitivity to doxorubicin. A,B) Sensitivity of DDRGK1‐knockout cells to DOX. The controlled and DDRGK1‐knockout cells were subjected to DOX(0.8 µg mL^−1^) for 24 h, then loaded with a DHE probe for 30 min and detected ROS level by flow cemetery. C,D) NRF2 overexpression rescues the ROS accumulation induced by DOX. cells were subject to DOX(0.8 µg mL^−1^) for 24 h and ROS levels were detected by flow cemetery. E) Half‐maximal inhibitory concentration (IC_50_) of doxorubicin in the controlled and DDRGK1‐knockout 143B cells detected by CCK8 assays. F,G) Flow cemetery to detect cell apoptosis by Annexin V/DAPI staining in the controlled and DDRGK1‐knockout 143B cells in the presence or absence of DOX (0.8 µg mL^−1^). H) Protein levels of cleaved caspase 3 and cleaved PARP in the controlled and DDRGK1‐knockout 143B cells in the presence or absence of DOX (0.8 µg mL^−1^) detected by western blot. I,J) Flow cytometry to analyze cell apoptosis by Annexin V/DAPI staining in controlled cells, DDRGK1‐knockout cells, and Nrf2 overexpressed DDRGK1‐knockout cells in the presence of DOX (0.8 µg mL^−1^). K,L) Flow cemetery to detect cell apoptosis by Annexin V/DAPI staining in the controlled and DDRGK1‐knockout 143B cells in the presence or absence of KI696. (MFI, mean fluorescence intensity; Ten samples for each statistical analysis, *p < 0.05; **p < 0.01; ***p < 0.001,****p < 0.0001).

DOX is one of the most commonly used chemotherapy drugs, and one of its mechanisms of action is the regulation of redox homeostasis via the NRF2 pathway.^[^ ^36^ ^]^ We speculated that the deletion of DDRGK1 might enhance DOX‐induced ROS accumulation through the regulation of NRF2, thus enhancing the sensitivity of OS cells to DOX. Similar to H_2_O_2_‐induced ROS, after cells were subjected to DOX for 24 h, intracellular ROS levels were elevated, as expected, and DDRGK1 knockout cells showed enhanced ROS accumulation (**Figure** **7**A,B). NRF2 overexpression rescued the elevated ROS levels resulting from the DDRGK1 knockout (Figure 7C,D). DOX led to concomitant dose‐dependent cellular death and a decrease in the half‐maximal inhibitory concentration (IC_50_) in DDRGK1 knockout cells (Figure 7E), suggesting that DDRGK1 knockout enhances tumor sensitivity to DOX. We then treated cells with etoposide, another chemotherapeutic agent similar to DOX that exerts an anti‐tumor effect by inhibiting topoisomerase II, and observed the same enhancement of chemosensitivity (Figure S11, Supporting Information). In addition, DDRGK1 knockout increased the rate of apoptosis as well as the levels of cleaved caspase 3 and cleaved PARP during DOX treatment (Figure 7F–H). We also observed a similar enhancement of chemosensitivity to DOX after NRF2 inhibition by an NRF2 inhibitor (Figure S12, Supporting Information), indicating that DDRGK1‐knockout‐induced chemosensitivity enhancement may downregulate NRF2 expression. Indeed, NRF2 overexpression rescued DOX‐induced cell death (Figure 6I,J), confirming that the loss of NRF2 was responsible for DDRGK1‐knockout‐induced cell death upon DOX treatment. Furthermore, KI696 also rescued DOX‐induced cell death in DDRGK1 knockout cells (Figure 7K,L), further demonstrating that the regulation of NRF2 by DDRGK11 affects the binding of KEAP1 and NRF2.

### 2.8 DDRGK1 Knockout Inhibits Osteosarcoma Tumorigenicity and Enhances Chemotherapy Sensitivity to Doxorubicin In Vivo

**Figure 8:**
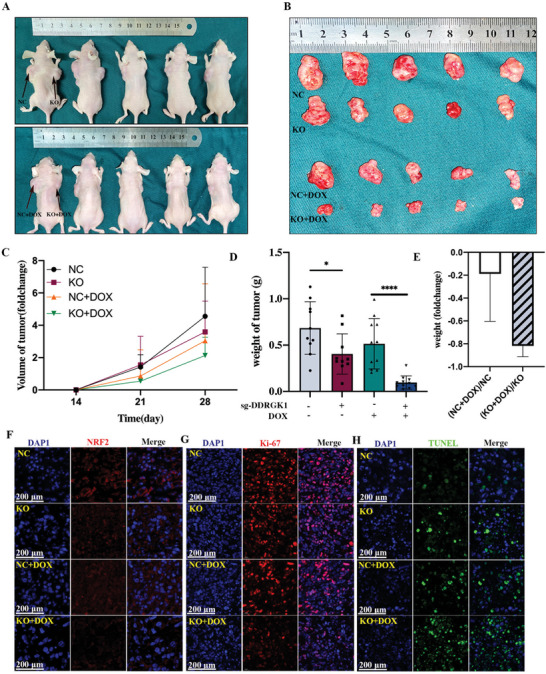
DDRGK1 knockout inhibits osteosarcoma growth in vivo A) BALB/c nude mice were transplanted with controlled cells at the left back and DDRGK1‐knockout 143B cells at the right back. Two weeks later doxorubicin (15 mg kg^−1^) was administered via intraperitoneal injection (*n* = 10). After another 2 weeks, the mice were sacrificed (*n* = 10). B) Tumor tissues dissected from mice at 4 weeks after transplantation. C) Tumor size measured every week after being treated with doxorubicin. D) Tumor weight measured at 4 weeks after transplanted. E) Relative decrease of tumor weight in NC + DOX, and KO + DOX groups. F) Immunofluorescence staining of Nrf2 in different tumor tissues. G) Immunofluorescence staining of Ki‐67 in different tumor tissues. H) Immunofluorescence staining of TUNEL in different tumor tissues. (*p < 0.05; **p < 0.01; ***p < 0.001,****p < 0.0001).

To examine the effects of DDRGK1 knockout on OS growth in vivo, normal 143B cells and DDRGK1 knockout cells were transplanted subcutaneously into BALB/c nude mice, and one dose of DOX was administered 2 weeks after transplantation. DDRGK1 knockout significantly decreased OS growth rate in terms of tumor size and weight when compared to control mice (**Figure** **8**A–D). Consistent with our previous results, DDRGK1 knockout displayed a stronger antitumor effect following DOX treatment in comparison to the control mice. An immunofluorescence assay was used to further assess the protein markers of interest. Initially, NRF2 decreased after DDRGK1 knockout with a further decrease evident following treatment with DOX (Figure 8E). Ki67, which represents cell proliferation, was also decreased (Figure 8F). TUNEL staining, which represents cell apoptosis, was elevated in DDRKG1 knockout tumors and was altered more remarkably after DOX treatment (Figure 8G). Additionally, we investigated whether DDRGK1 overexpression promotes OS growth. To test this hypothesis, 143B cells stably expressing DDRGK1 were transplanted subcutaneously. As expected, tumors derived from cells overexpressing DDRGK1 were larger than those in the control (Figure S13A–D, Supporting Information), further supporting the idea that DDRGK1 may play a role in oncogenesis. These data suggest that knocking out DDRGK1 inhibits OS tumorigenicity and enhances sensitivity to DOX, thus providing a new potential therapeutic target for OS.

## 3 Discussion

**Figure 9:**
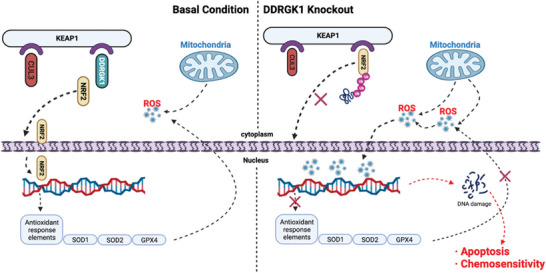
A schematic diagram of regulation of NRF2 activity by DDRGK1. At basal conditions, DDRGK1 binds competitively to the Kelch domain of KEAP1 and stops NRF2 ubiquitin‐mediated degradation, allowing its nuclear translocation, thus exerting its role as a transcription factor and promoting transcription of ARE genes to eliminate ROS. When DDRGK1 is knocked out, the inhibition of NRF2 binding to KEAP1 is abolished thereby promoting its ubiquitination and impairing the cell antioxidant capacity, leading to cell apoptosis and chemosensitivity induced by ROS‐mediated DNA damage.

In the present study, we demonstrated that DDRGK1 promoted OS cell growth and positively correlated with poor clinical prognosis. Furthermore, quantitative proteomics‐based enrichment analyses indicated that DDRGK1 was involved in multiple mitochondrial activities. Interestingly, the DDRGK1 knockout induced ROS accumulation. It regulated redox homeostasis by competing with the ROS key protein NRF2 to bind KEAP1, which recruits CUL3 to tag NRF2 with ubiquitin. Thus, DDRGK1 inhibited the E3‐ubiquitin‐ligase‐mediated proteasomal degradation of NRF2. Deletion of DDRGK1 abolished the suppression of KEAP1 and promoted NRF2 degradation, thereby disturbing the cellular antioxidation system. The absence of DDRGK1 correlated with ROS accumulation and enhanced chemosensitivity to DOX and etoposide in OS cancer cells. Tumor growth was significantly reduced in DOX‐treated DDRGK1 knockout mice compared to that in wild‐type mice, indicating a promising novel clinical strategy for OS treatment (**Figure** **9**).

Resistance to chemotherapy remains a major challenge in enhancing the OS survival rate. ROS levels and antioxidant protein activity are usually upregulated in tumor cells, especially in chemoresistant tumor cells. This makes cancer cells more vulnerable to alterations in the ROS levels.^[^ ^15^, ^16^ ^]^ Conventional chemotherapies, such as DOX and etoposide, largely kill cancer cells by augmenting ROS‐induced stress.^[^ ^6^ ^]^ The direct mechanism of action of both DOX and etoposide is via inhibition of DNA topoisomerase II activity,^[^ ^37^, ^38^ ^]^ with many studies reporting that ROS plays an important role in triggering apoptosis of cancer cells through the mitochondrial pathway.^[^ ^39^, ^40^ ^]^ The effect of ROS in these chemo‐drugs seems paradoxical; mild or chronic ROS trigger chemoresistance, while modulating ROS by pharmacological or genetic interventions simultaneously also enhances drug sensitivity.^[^ ^8^ ^]^ Thus, in order to achieve effective anti‐tumor effects and avoid chemoresistance, sufficient or even excessive doses must be applied in clinical practice, which also leads to dose‐dependent toxicity to normal cells. Fortunately, there have been successful attempts to enhance chemosensitivity via ROS modulation. For example, natural borneol potentiates DOX‐induced G2/M cell cycle arrest by triggering ROS‐mediated DNA damage in human glioma cells.^[^ ^41^ ^]^ The combination of panobinostat and etoposide induces strong cell death responses in cervical cancer‐derived cells through increased ROS production.^[^ ^39^ ^]^ These data indicate that modulating ROS levels is an effective method for targeting and sensitizing chemoresistant cancer cells.

Accumulating evidence has established the key ROS sensor NRF2 as a driver of cancer progression and metastasis, which also confers chemoresistance.^[^ ^42^, ^43^, ^44^ ^]^ Using the N‐terminus of its basic leucine zipper domain, NRF2 can bind to the NF‐E2 site in the DNA‐globin gene cluster, which has been identified as an enhancer region encoding antioxidant proteins.^[^ ^32^, ^45^ ^]^ Downregulation of NRF2 can significantly disrupt the global redox balance, resulting in uncontrolled ROS accumulation. Several reports have shown that NRF2 activation promotes tumor chemoresistance and indicates that it is a promising target for overcoming ROS‐dependent chemotherapy resistance. For example, PAQR4 physically interacts with NRF2 and blocks the interaction between NRF2 and KEAP1, enhancing the sensitivity of cancer cells to chemotherapy.^[^ ^46^ ^]^ Overexpression of NRF2 renders NH3 cells resistant to GPX4 inhibitors by promoting ferroptosis.^[^ ^47^ ^]^ In this study, we identified DDRGK1 as a novel regulator of NRF2. Several studies have suggested that NRF2 has various novel functions, particularly in cell proliferation and apoptosis, and NRF2 has been considered to control these cellular processes by regulating the cellular levels of ROS. These phenomena are consistent with the observation that the DDRGK1 knockout affects cell viability and apoptosis. This is also in accordance with clinical data showing that higher NRF2 expression correlates with a lower survival rate.^[^ ^48^ ^]^

NRF2 stability is key to ROS homeostasis and is mainly degraded via the ubiquitin‐proteasome pathway.^[^ ^33^ ^]^ KEAP1 acts as an adaptor to bridge NRF2 with a CUL3‐based E3 ligase. Therefore, disrupting the interaction between NRF2 and either KEAP1 or CUL3 may influence NRF2 degradation. For example, iASPP competes with NRF2 for KEAP1 binding via a DLT motif, leading to decreased NRF2 ubiquitination.^[^ ^49^ ^]^ Another antioxidative protein, FAM129B, competes with NRF2 for KEAP1 binding via its DLG and ETGE motifs.^[^ ^50^ ^]^ Similar to these studies, we also found that DDRGK1 can competitively bind to the Kelch domain of KEAP1, thus blocking the interaction of NRF2 with CUL3 and inhibiting NRF2 degradation. DDRGK1 deletion induces NRF2 protein degradation and decreases the expression of downstream antioxidant proteins, thereby inducing ROS production. Consistently, previous studies found that stable knockdown of DDRGK1 results in ER‐phagy, and mitochondrial oxidative phosphorylation also promotes ER‐phagy; thus, a linkage appears to exist between DDRGK1 and mitochondrial oxidative phosphorylation.^[^ ^51^ ^]^ In our study, we observed a remarkable enhancement in oxidative phosphorylation and mitochondrial metabolism after DDRGK1 knockout. The accumulated ROS might stem from activated mitochondrial metabolism, while the underlying mechanism linking DDRGK1 and mitochondria needs further exploration. In addition, we must note that the reported phenotypes on DDRGK1 knockout may not be entirely dependent on NRF2. In fact, DDRGK1 plays versatile roles in biological processes, such as regulation of IRE1*α* stability,^[^ ^12^ ^]^ ASC1 UFMylation,^[^ ^30^ ^]^ SOX9 ubiquitination,^[^ ^16^ ^]^ and so on. It remains unclear to what extent NRF2 activity contributes to the DDRGK1 knockout phenotype. Nevertheless, our work revealed the role of DDRGK1 in the regulation of NRF2 expression and provided an incremental advance in understanding DDRGK1.

In conclusion, the current study demonstrates an unprecedented role for DDRGK1 in maintaining redox homeostasis via the regulation of the KEAP1/NRF2 pathway. DDRGK1 deletion significantly enhanced chemosensitivity, providing a novel therapeutic strategy to overcome chemoresistance in OS treatment.

## 4 Experimental Section

### Bioinformatics

Pan‐cancer gene expression analysis using the TCGA database was performed using TCGAbiolinks and edgeR R packages. Only tumors with more than 10 paired normal tissues were selected for analysis. The DepMap database, a large‐scale loss‐of‐function screening database using CRISPR‐Cas9 and RNAi, was used to evaluate the role of DDRGK1. The score evaluated the size of the effect of knocking out or knocking down a gene while normalizing expression against the distribution of pan‐essential and nonessential genes.^[^ ^25^, ^52^ ^]^ A score of 0 was equivalent to a gene that is not essential, whereas a score of −1 corresponded to the median of all common essential genes. A negative score meant that gene knockdown inhibited cell growth. GEO data were downloaded using the GEOquery R package. GSE18043, GSE38718, and GSE14827 were used to compare DDRGK1 expression between MSCs, muscle, and OS tissues. The GSE21257 dataset was used for survival analysis using the “survminer” R package and patients’ baseline data were shown in Table S1, Supporting Information.

### Cell Culture, Reagents, and Vectors

Saos‐2, U2OS, MG63, Fhob1.19, L6, and HEK293T cells were purchased from the cell bank of the Chinese Academy of Science (Shanghai, China). 143B cells were purchased from the American Type Culture Collection (ATCC). Except for the Saos‐2 cells that were cultured in McCoy's 5A Medium (Gibco, USA), all other cells were cultured in Dulbecco's modified Eagle medium (DMEM) (HyClone, USA) supplemented with 10% fetal bovine serum (FBS; VWR, USA) and 100 U mL^−1^ penicillin/streptomycin (Gibco, USA). All cell lines were maintained in an incubator (Thermo Fisher Scientific, USA) with 5% CO_2_ at 37 °C. Cycloheximide, DOX, TBHq, and brusatol were purchased from Selleck (Shanghai, China). KI696 was purchased from MedChemExpress (China). The DDRGK1 shRNA and sgRNA plasmids, DDRGK1‐flag plasmid, DDRGK1‐Myc‐plasmid, NRF2‐HA plasmid, KEAP1‐HA plasmid, KEAP1‐HA truncation plasmid (amino acids 1–320321‐644), DDRGK1‐Flag truncation plasmid (amino acids 1–210, 210–280, 280–314), and Cas9 lentivirus were constructed by IBSBio (Shanghai, China).

### Plasmid and Lentiviral Transfections

To transfect cells with shDDRGK1, DDRKG1‐Myc, DDRGK1‐Flag, KEAP1‐HA, NRF2‐HA, ubiquitin‐myc plasmids, and 5 µg of the target plasmid was mixed with 10 µL of lipofectamine 3000 and 10 µL of P3000 for 15 min and then added to cells for a 48‐h transfection. Lentiviruses were generated by transfecting 1.5 µg of pMD2.G, 4.5 of µg psPAX2, and 6 µg of target plasmids, including sgDDRGK1‐puro or NRF2‐HA‐puro, into HEK293T cells. The virus‐containing supernatant was collected after a 48‐h transfection, and the target cells were transfected by polybrene for 48 h followed by a stable transfected cell selection using puromycin. The DDRGK1 knockout cells were constructed by a two‐step transfection; briefly, Cas9 lentiviruses were transfected, followed by sgDDRGK1 lentiviral transfection. The doxycycline‐inducible DDRGK1 knockdown system was performed by transfected with pLKO‐Tet‐On‐shDDRGK1 lentivirus (IBSBio, Shanghai, China) in 143B cells and 100 ng mL^−1^ doxycycline was applied for 48 h to induce shRNA expression and DDRGK1 protein knock‐down.^[^ ^53^ ^]^

### Cell Viability Assay

Cell viability was tested using a Cell Counting Kit (CCK8, Beyotime, China). The same density of cells was seeded into a 96‐well plate and cultured for 24 h with or without stimulators (DOX 1 µg mL^−1^, Tg 10 nM) treated for indicated time points. Then, 10 µL of CCK8 reagent was added, maintained at 37 °C for 1 h. The optical density was then measured at 450 nm using an automatic i‐control microplate reader (Tecan Life Sciences, Switzerland).

### Colony Formation Assay

Indicated cells were seeded into a 6‐well plate at a density of 800 cells per well and cultured for about 5–7 days according to the size of the colony observed using an inverted microscope. Then cells were fixed using 4% paraformaldehyde for 30 min and stained for 2 h with 0.1% crystal violet solution (Solarbio, Beijing, China). Positively stained colonies were imaged and quantitated by the ImageJ image processing program (NIH, USA).

### Cell Cycle Assay

Indicated cells were seeded into a 12‐well plate and cultured in DMEM without FBS for 24 h. The media was then changed with DMEM containing 10% FBS, and cells were cultured for another 24 h. Next, cell suspensions were prepared, incubated with 0.5 µg mL^−1^ DAPI (Beyotime, China) for 10 min, and subjected to flow cytometry on an LSRFortessa flow cytometer (BD Biosciences, USA) counting at least 10 000 events. Data were processed using FlowJo software (Version 10.4, BD Biosciences, USA).

### Cell Apoptosis Assay

Cells were seeded into a 12‐well plate and treated with indicated drugs for 24 h (DOX 1 µg mL^−1^, Tg 0.1 uM) for 24 h. Then, cells were harvested and examined by LSRFortessa flow cytometer (BD Biosciences, USA) after staining with the Annexin V‐mCherry Kit (Beyotime Biotechnology, China) and 0.5 µg mL^−1^ DAPI (Beyotime Biotechnology, China). Data were processed using FlowJo software (BD Biosciences, USA).

### Proteomics Analysis

DDRGK1 knockout 143B cells and control cells were seeded in a T75 dish. Whole‐cell protein extracts were harvested using lysis buffer under ultrasound (three replicates in each group). The solutions were collected and lyophilized. For TMT labeling, the lyophilized samples were resuspended in 100 µL of 200 mM TEAB (Thermo Fisher Scientific, USA) and 40 µL of each sample was mixed for labeling. Samples were then desalted using a C18‐Reverse‐Phase SPE Column (Thermo Fisher Scientific, USA). Phosphopeptides were enriched from samples using titanium dioxide beads (TiO_2_) according to a modified protocol from Jersie‐Christensen et al.^[^ ^54^ ^]^ All analyses were performed using a Q‐Exactive mass spectrometer (Thermo Fisher Scientific, USA) equipped with a Nanospray Flex source (Thermo Fisher Scientific, USA). Samples were loaded and separated using a C18 column (15 cm × 75 µm) on an EASY‐nLC TM 1200 system (Thermo Fisher Scientific, USA). The flow rate was 300 nL min^−1^ and the linear gradient was 60 min (0–40 min, 5–30% B; 40–54 min, 30–50% B; 54–55 min, 50–100% B; 55–60 min, 100% B; mobile phase. A = 0.1% FA in water and B = 80% ACN/0.1% FA in water). Full MS scans were acquired in the mass range of 300–1600 m/z with a mass resolution of 70 000, and the AGC target value was set at 1e6. The 10 most intense peaks in MS were fragmented with higher‐energy collisional dissociation (HCD) with an NCE of 32. MS/MS spectra were obtained with a resolution of 17 500 with an AGC target of 2e5 and a max injection time of 80 ms. The Q‐E dynamic exclusion was set for 30 s and run in positive mode. Proteome Discoverer (v.2.4) was used to search all of the raw data thoroughly against the sample protein database. The database search was performed with trypsin digestion specificity. Alkylation on cysteine was considered a fixed modification during database searching. For protein quantification, the TMT labeling method was selected. A global false discovery rate was set at 0.01, and protein groups considered for quantification required at least two peptides. The protein expression values were evaluated by Proteome Discover software (Thermofisher, 2.4). The mass spectrometry proteomics data have been deposited to the ProteomeXchange Consortium (http://proteomecentral.proteomexchange.org) via the iProX partner repository^[^ ^55^ ^]^ with the dataset identifier PXD035473.

### Mitochondrial Respiratory Capacity Assay

Cells were seeded in an XF microplate at an optimal density and cultured for 24 h. The cellular oxygen consumption rate (OCR) was measured using a Seahorse XF Analyzer (Agilent, USA) following the manufacturer's protocol. Briefly, basal respiration was first detected, then 1 µM of ATP synthase inhibitor oligomycin was added and ATP production was detected. Next, 2 µM FCCP was injected to detect maximal respiration, and 5 µM of antimycin A and rotenone was added to detect spare respiration capacity. All the reagents and plates were purchased from Agilent (USA).

### Intracellular ROS Detection

Cells were treated with H_2_O_2_ for 20 min or DOX for 24 h, followed by incubation with 10 µM of DHE probe (Biolab, China) for 30 min at 37 °C. Cells were then suspended in serum‐free medium with 0.1 µg mL^−1^ DAPI and stained for 10 min followed by flow cytometry analysis. Mean fluorescence intensity (MFI) was used to evaluate ROS level.

### Quantitative Reverse Transcription‐PCR

Total RNA was harvested using a Trizol reagent (Ambion, USA) and quantified by a UV spectrophotometer. 1000 ng of RNA was mixed with 4 µL of RT Reagent Kit (Takara, Japan) and incubated in a Veriti 96‐Well Thermal Cycler (Thermo Fisher Scientific, USA). Next, ≈4.4 µg of cDNA mixed with 0.4 µg primer and 5 µL of TB Green Premix Ex Taq Kit (Takara, Japan) were used for each PCR reaction, performed with TB Green (Takara, Japan) in a QuantStudio 6 Flex Real‐Time PCR System (Thermo Fisher Scientific, USA). Gene expression relative to ß‐actin was calculated using the comparative 2^−ΔΔCT^ method. Primer sequences used are listed in Table S2, Supporting Information.

### Immunoblotting and Co‐Immunoprecipitation Assay

For immunoblotting, whole cell extracts were lysed for 20 min at 4 °C using a RIPA lysis buffer (Beyotime, China) containing 1% protease and phosphatase inhibitor cocktail. They were then mixed with SDS‐PAGE loading buffer (Biosharp, China) and boiled at 100 °C for 5 min. Solubilized proteins were subjected to SDS‐PAGE and western blot analysis. For co‐IP, whole cell extracts were dispersed in 1 mL of cell lysis buffer (Beyotime, China) containing 1% protease inhibitor cocktail at 4 °C for 20 min, and then Anti‐Myc or Anti‐Flag or Anti‐HA immunomagnetic beads (Biomake, China) were added into solubilized proteins and waved at 4 °C overnight. On the second day, the immunomagnetic beads were collected by a magnet, washed three times, and then mixed with loading buffer and boiled at 100 °C for 5 min, finally followed by an immunoblotting procedure. Antibodies against DDRGK1, NRF2, KEAP1, and CUL3 were purchased from ProteinTech (China), the ß‐actin antibody was purchased from Affinity (USA), and PI3K, phosphorylated PI3K, AKT, phosphorylated AKT, cleaved caspase 3, and the PARP antibody were purchased from Cell Signaling Technology (USA).

### Protein Expression and Purification

The Kelch domain (residues 312–624) of human KEAP1 (His tagged) and N‐terminal domain (residues 1–84) of human NRF2 (GST tagged) were expressed and purified as described previously.^[^ ^56^ ^]^ Briefly, plasmids were transformed and expressed in BL21 (DE3) competent Escherichia coli cells. Cells were then lysed and applied to a Ni‐NTA column or GSH column. Next, the proteins were applied for further purification via gel filtration chromatography. Human Fc‐tagged DDRGK1 and Fc proteins were expressed in HEK293F cells and purified via Protein G Resin. The human KEAP1, NRF2 plasmids, and Fc‐DDRGK1 and Fc plasmids were synthesized and constructed by Tsingke Biotechnology Co., Ltd.

### Pulldown Experiments

For Fc pulldown, recombinant Fc‐DDRGK1 and Fc proteins were incubated with Protein A/G immunomagnetic beads (Biomake, China) at room temperature for 0.5 h, then mixed with recombinant NRF2 and KEAP1 proteins at 4 °C overnight. On the second day, the immunomagnetic beads were collected by a magnet, washed three times, and then mixed with loading buffer and boiled at 95 °C for 5 min, finally followed by an immunoblotting procedure. For GST pulldown or His pulldown, recombinant GST‐NRF2 protein or His‐KEAP1 protein was incubated with GST immunomagnetic beads (Beyotime, China) or His immunomagnetic beads (Beyotime, China) at room temperature for 0.5 h, then mixed with other indicated recombinant proteins at 4 °C overnight. On the second day, the immunomagnetic beads were collected by a magnet, washed three times, and then mixed with loading buffer and boiled at 95 °C for 5 min, finally followed by an immunoblotting procedure.

### Immunofluorescence and Confocal Microscopy Analysis

To observe the localization of DDRGK1 and KEAP1, 143B cells were seeded in a confocal petri dish at a density of 200 × 10^3^ per well and fixed with 4% paraformaldehyde for 30 min, then blocked by 5% BSA solution for 1 h. Later, cells were conjugated with anti‐rabbit‐DDRGK1 and anti‐mouse‐KEAP1 antibodies overnight at 4 °C. The next day, cells were incubated with goat anti‐rabbit IgG H&L (Alexa Fluor 488) and goat anti‐mouse IgG H&L (Alexa Fluor 555) at room temperature for 1 h, then stained with DAPI for another 10 min. Finally, cells were observed using a Leica confocal microscope, and co‐localization correlation analysis was performed using Fiji software.

### Immunohistochemistry

The OS tissue array was purchased from Biomax (CAT:OS804d, USA). The array slide was deparaffinized and dried before antigen retrieval. Next, goat serum was applied to the blocking antibody and incubated for 20 min, then incubated with the Anti‐DDRGK1 antibody overnight at 4 °C. The next day, a biotin‐conjugated secondary antibody was applied at room temperature for 1 h, followed by incubation with SABC reagent at 37 °C for 20 min. Finally, the slide was treated with chromogen from the final developmental DAB kit and observed using an inverted microscope.

To compare the expression of DDRKG1 in different tissues, the DAB positive area and integrated optical density (IOD) were calculated using ImageJ software and protein expression was represented by mean density (IOD/area).

### Mouse Xenograft Model

The nude mice were fed with routine food, and 1 × 10^6^ 143B wild‐type cells and DDRGK1‐knockout cells suspended in DMEM medium were injected subcutaneously into their backs (4 W, Bikai, China). After 2 weeks, a DOX solution was intraperitoneally injected (15 mg kg^−1^ per mouse). Then, tumor volume was measured every week with a vernier scale. Two weeks later, the nude mice were executed, tumor tissues were excised, and tumor weight and volume measurements were made. The solid tumors were then fixed using 4% paraformaldehyde for 48 h and sliced for immunofluorescence staining. The study was approved by laboratory animal ethics committee of Shanghai Ninth People's Hospital (Approval No:SH9H‐2022‐A91‐1).

### Statistical Analysis

Data were presented as a mean ± standard error from three independent experiments. Student's *t*‐test was applied to assess the statistical significance. *p* values < 0.05 were considered significant. At least three samples were included in each statistical analysis. The Kaplan–Meier method was used to compare overall survival between different groups. Statistical analyses were performed using GraphPad Prism software (version 9.0.0, USA). Bioinformatics analyses were performed using R Project for Statistical Computing (version 4.1.3).

## Conflict of Interest

The authors declare no conflict of interest.

## Supporting information

Supporting Information

## Data Availability

The data that support the findings of this study are available from the corresponding author upon reasonable request.
