# Supplementary material for: DDRGK1 Enhances Osteosarcoma Chemoresistance via Inhibiting KEAP1‐Mediated NRF2 Ubiquitination
Source: Adv Sci (Weinh). 2023 Mar 25;10(14):2204438. doi: 10.1002/advs.202204438 (PMC10190621; doi:10.1002/advs.202204438)
Supplement: Supplementary file 1 — Supporting Information [file ADVS-10-2204438-s001.pdf]

Supporting Information

DDRGK1 promotes osteosarcoma chemoresistance via inhibiting KEAP1-mediated NRF2 ubiquitination

Xin Wan[a], Tangjun Zhou, Xiao Yang, Xiankun Cao, Gu Jin, Pu Zhang,Jiadong Guo, Kewei Rong, Baixing Li, Yibin Hu, Kexin Liu, Peixiang Ma, An Qin, Jie Zhao

Supplementary Figures and Tables:

Contents

Figure S1 2
Figure S2 3
Figure S3 5
Figure S4 6
Figure S5 7
Figure S6 8
Figure S7 9
Figure S8 10
Figure S9 10
Figure S10 11
Figure S11 12
Figure S12 13
Figure S13 14

Figure S1

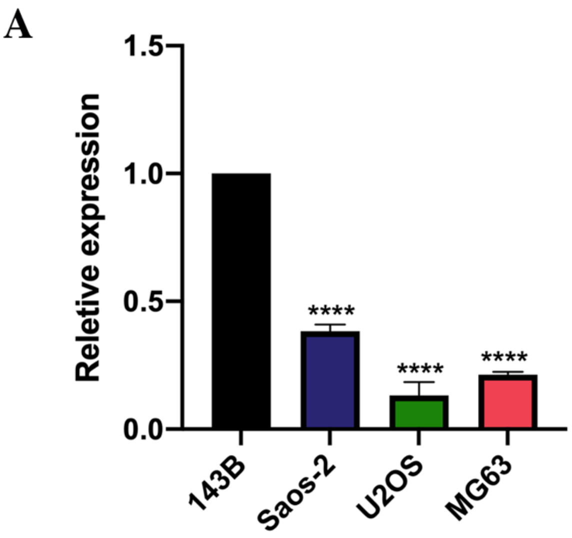

Figure S1. Comparing expression of DDRGK1 in different osteosarcoma cell lines. The 143B cell represents highest malignancy than Saos-2, U2OS and MG63 cells. The mRNA levels were detected by real-time quantitative PCR,β-actin is used as internal reference.

Figure S2

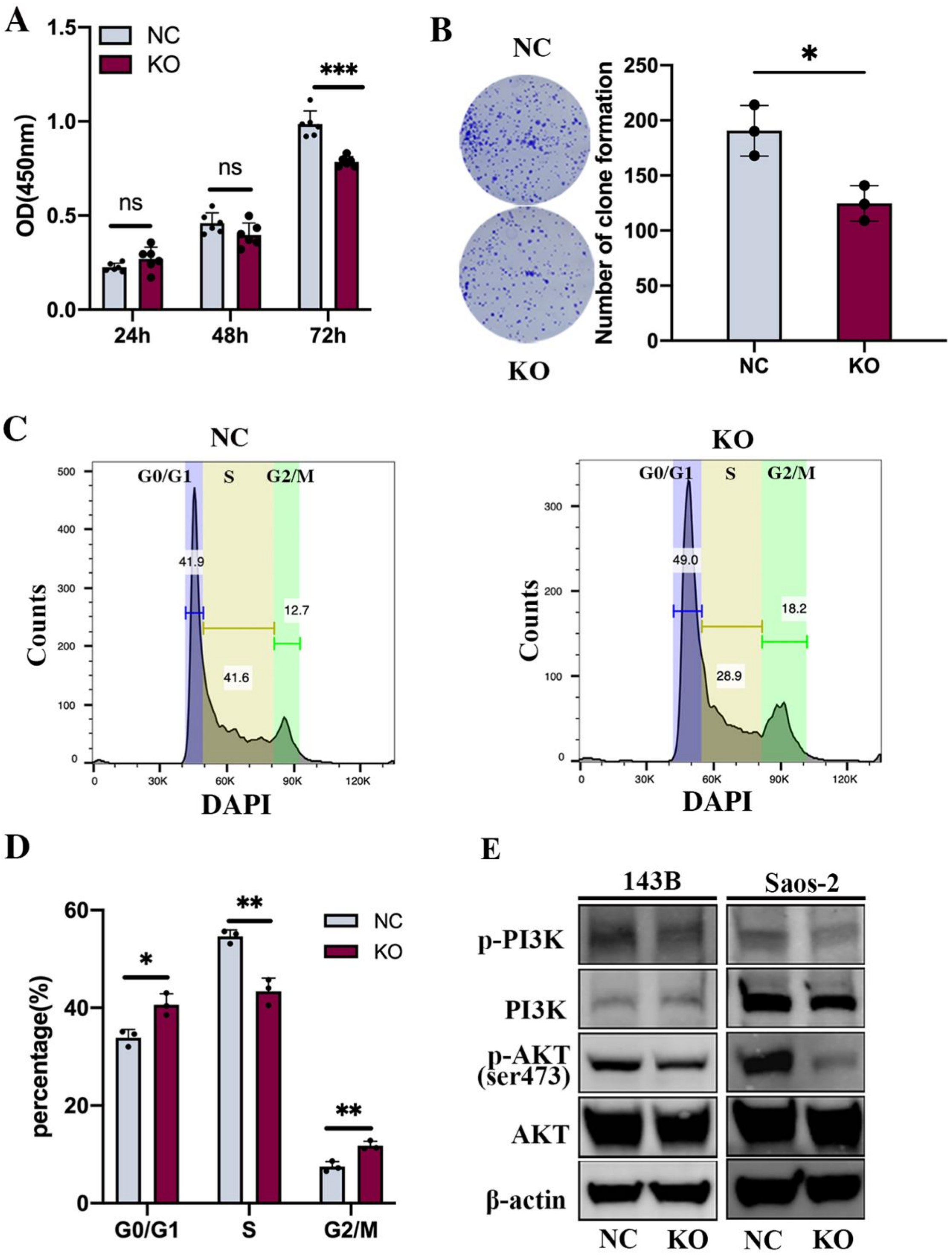

Figure S2. DDRGK1 knockout inhibits cell proliferation. (A) Wild-type Saos2 and DDRGK1-knockout Saos2 cells were cultured for indicated times and cell viability was detected by CCK-8 assay.(B) Determining the effect of DDRGK1 for cancer cells clone formation. Wild-type Saos2and DDRGK1-knockout Saos2 cells with 800 cells per well were

seeded in plate and cultured for 7 days, followed by crystal violet staining.(C-D) Determining the effect of DDRGK1 on cell cycle. Wild-type Saos2 and DDRGK1-knockout Saos2 cells were seeded in plate and hungered without serum for 24h, then cultured for another 24h with serum and collected for Flow cemetery analysis.(E) Protein levels of PI3K-AKT pathway detected by western blot in 143B and Saos-2 cells with or without deletion of DDRGK1.

Figure S3

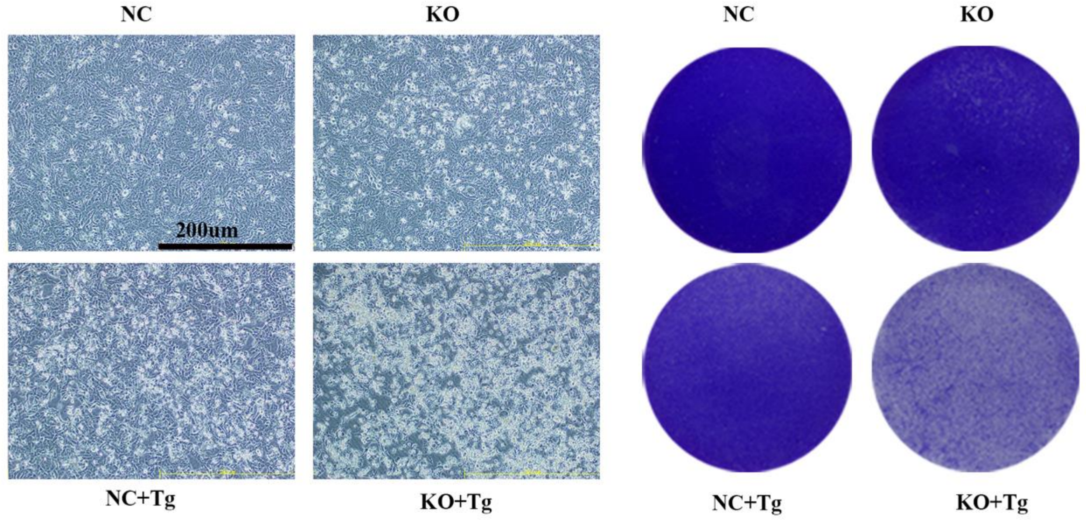

Figure S3. Determin the apopstosis induced by Tg. The controlled and DDRGK1-knockout 143B cells were cultureed and stimulated with or without Tg(20nM) for 24h. Cell morphology observed under inverted microscope and crystal violet staining to observe alived adherent cells.

Figure S4

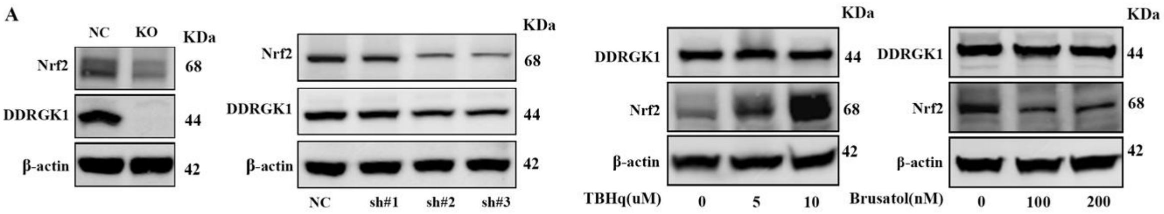

Figure S4. Identify the regulation between DDRGK1 and NRF2. (A) Regulation of NRF2 by DDRGK1. The expression of NRF2 in controlled cell and DDRGK1-knockout Saos-2 cells were detected by western blot. (B) Protein levels of NRF2 in HEK293T cells transfected by three sh-DDRGK1 plasmids detected by western blot. (C) Influence of DDRGK1 expression by NRF2 agonist. The 143B cell were treated with TBHq with different concentrations for 24h, and the DDRGK1 level was detected by western blot. (D) Influence of DDRGK1 expression by NRF2 inhibitor. The 143B cell were treated with Brusatol with different concentrations for 24h, and the DDRGK1 level was detected by western blot.

Figure S5

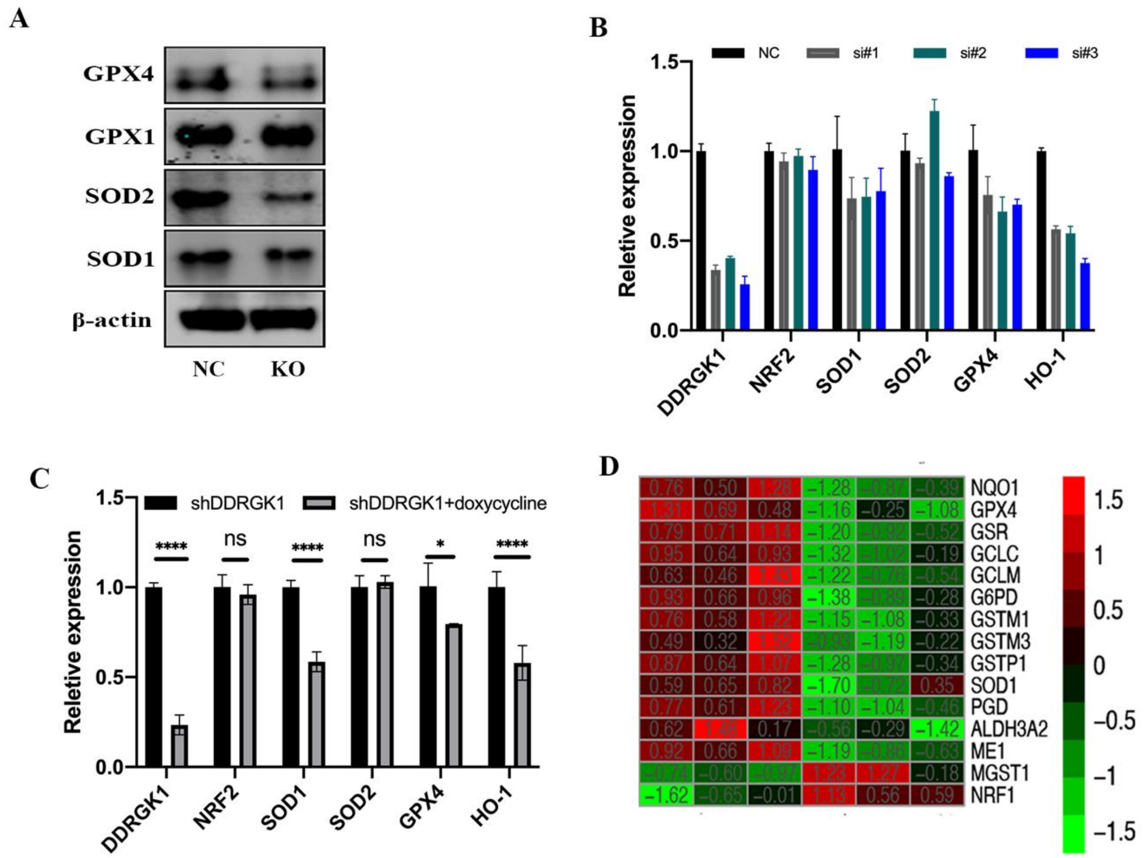

Figure S5. Influence of DDRGK1 on expression of antioxidant genes and proteins. (A) Protein levels of Nrf2 regulated downstream genes in in the control and DDRGK1-knockout Saos-2 cells. (B) mRNA levels in DDRGK1 acutely deleted cells by DDRGK1 siRNA. (C) mRNA levels in DDRGK1 acutely deleted cells by doxycycline-inducible system. (D) Heatmap for Nrf2 regulated downstream proteins in the control and DDRGK1 knockout 143B cells according to proteomics analysis.

Figure S6

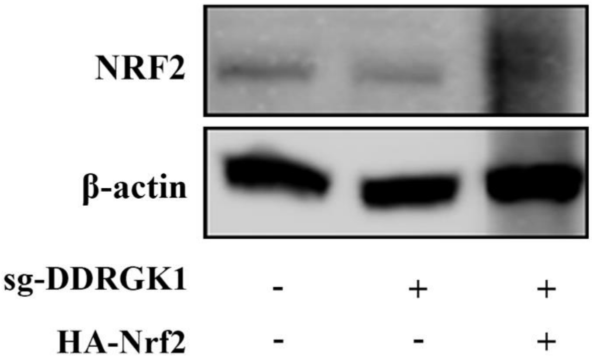

Figure S6. Identify overexpression of NRF2. The NRF2-HA plasimd was transfacted into DDRGK1-knockout 143B cells and the NRF2 level was detected by Western blot.

Figure S7

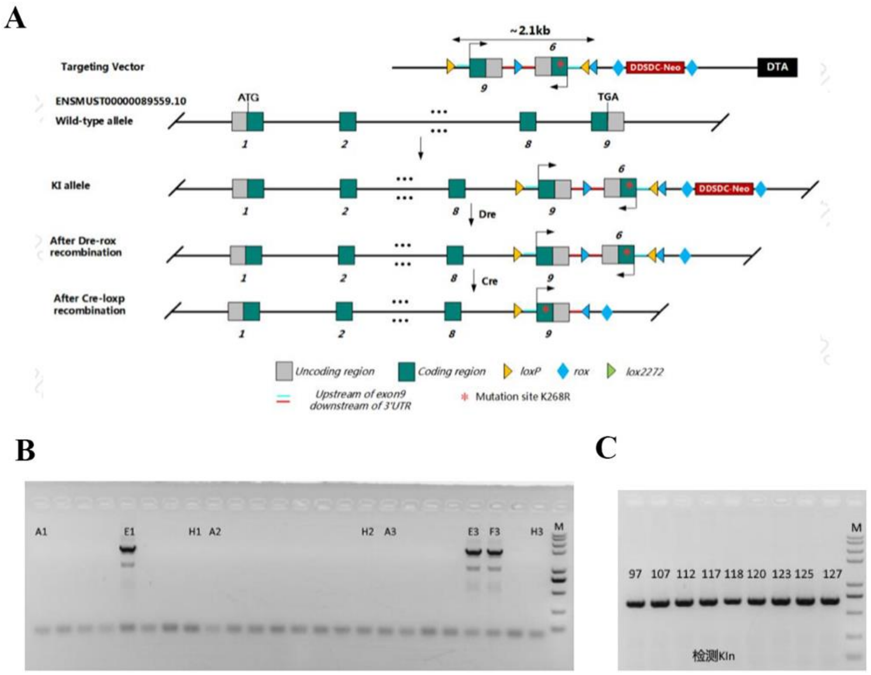

Figure S7: Construction of DDRGK1 K268R C57BL/6NGpt mouse. (A) The schematic diagram for K268R mutation conditional knock-in using Lox2272/wt system. (B) After K268R knocked into embryonic stem cell (ES cell), PCR was performed to verify its successful construction. E1,E3,F3 were identified as positive cells.(C) The F1 mice were born with their tails cut in 5-7 days. Genomic DNA was extracted for PCR to confirm genotypes. 97,107,112,117,118,120,123,125,127 were identified as positive mice and selected for subsequent reproduction.

Figure S8

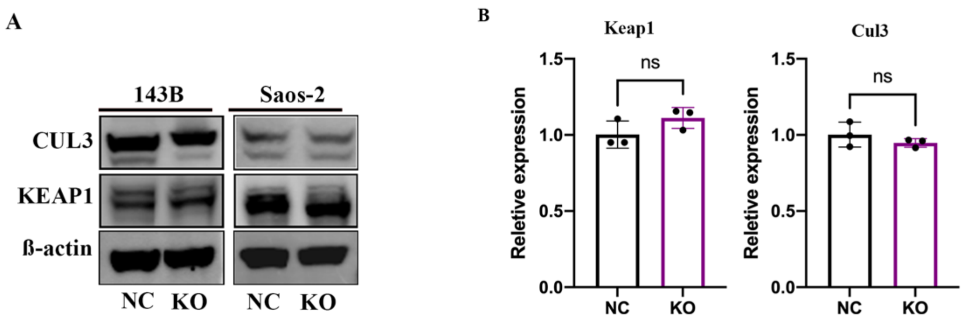

Figure S8. Influence of DDRGK1 knock on expression of KEA1 and CUL3. (A) protein levels of CUL3 and KEAP1 were detected by Western blot in 143B and Saos-2 cells with or without DDRGK1 knockout. (B) mRNA levels of CUL3 and KEAP1 were detected by Western blot in 143B cells with or without DDRGK1 knockout.

Figure S9

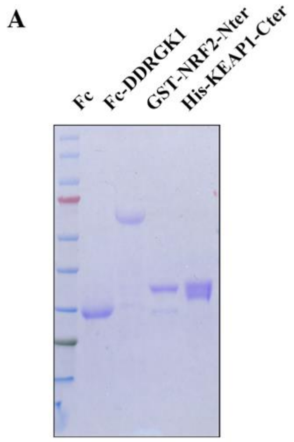

Figure S9. (A) Purified proteins of Fc, Fc-DDRGK1, GST-NRF2-Nter, His-KEAP1-Cter and stained by coomassie blue.

Figure S10

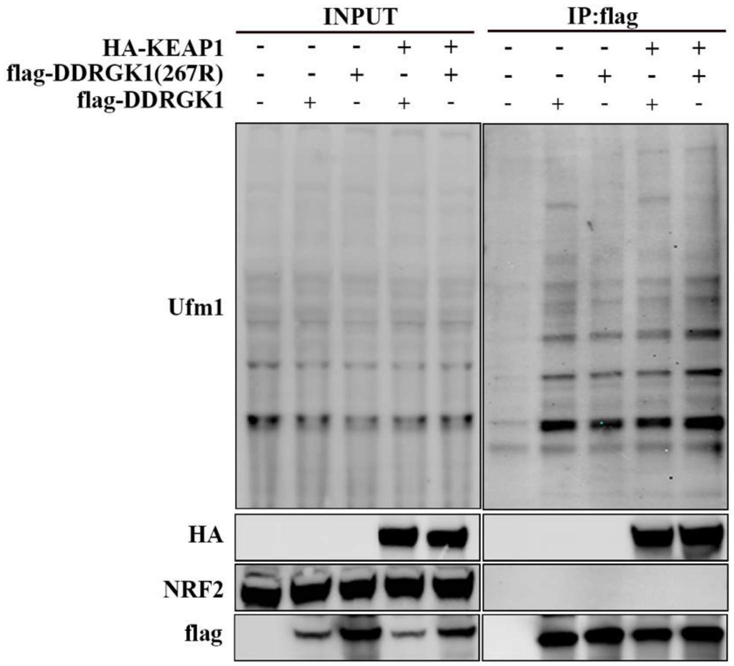

Figure S10. Co-IP assay to identify influence of DDRGK1 K267R mutation on interaction between DDRGK1 and KEAP1 in HEK293T cells. Cells were transfected with wildtype DDRGK1 or K267R mutation plasmid with or without co-transfected with HA- Keap1 plasmid.

Figure S11

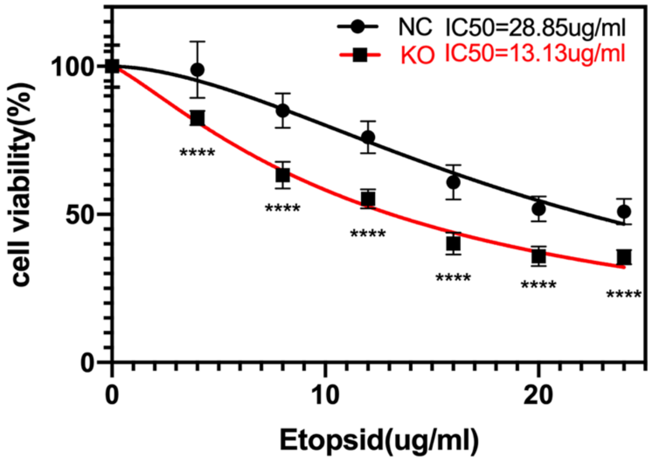

Figure S11. Half-maximal inhibitory concentration (IC_{50}) of Etoposid in the controlled and DDRGK1-knockout 143B cells detected by CCK8 assay.

Figure S12

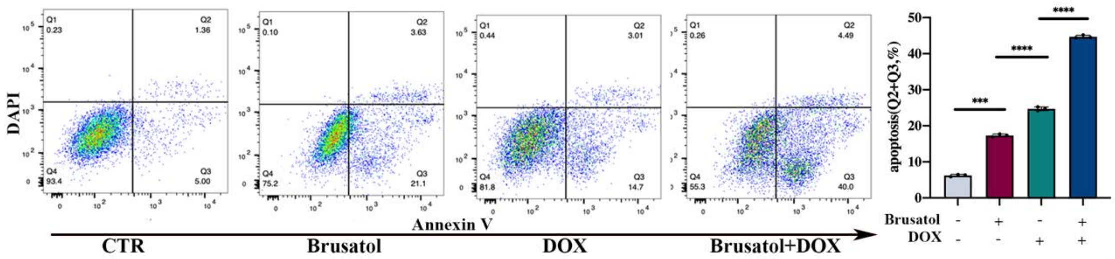

Figure S12. NRF2 inhibitor enhances cell sensitivity to DOX. 143B cells were treated with or without Brusatol(100nM) and DOX(0.8ug/ml) for 24h , then cell apoptosis was detected by Annexin V/DAPI staining.

Figure S13

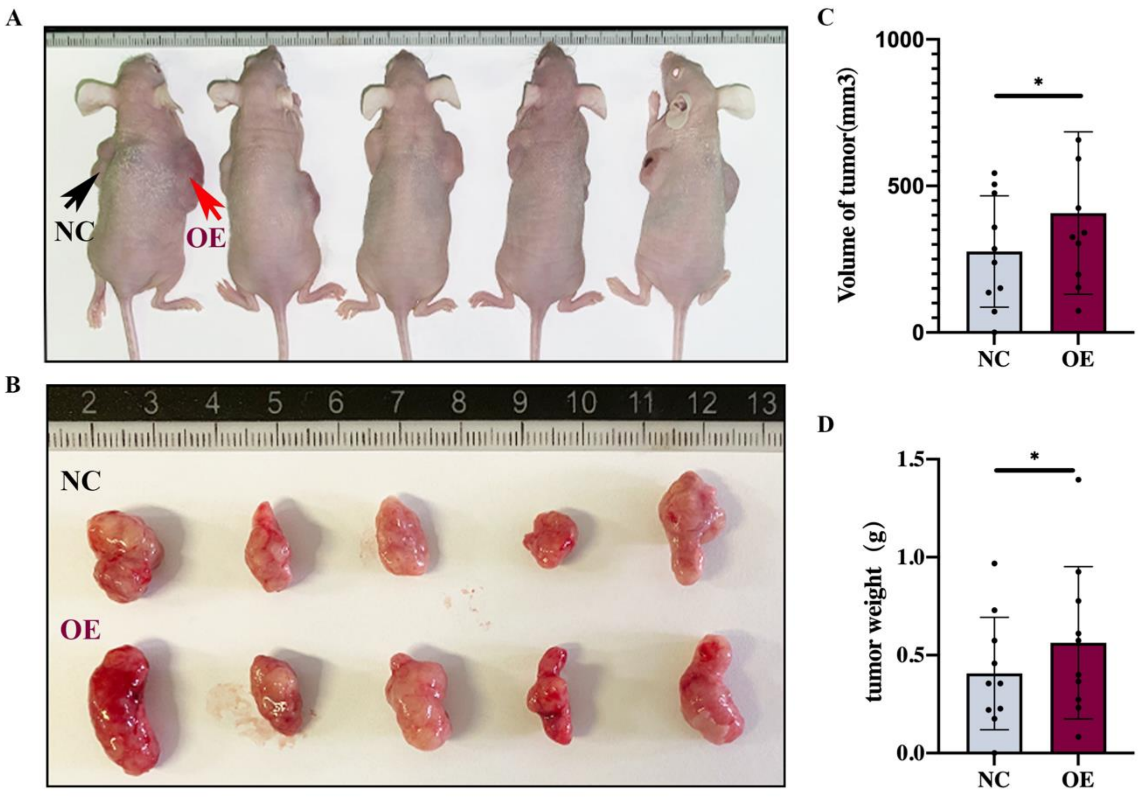

Figure S13. DDRGK1 overexpression promotes osteosarcoma growth in vivo (A) BALB/c nude mice were transplanted with controlled cells at left back and DDRGK1 overexpressed 143B cells at right back. After 4 weeks, the mice were sacrificed (n=10). (B) Tumor tissues dissected from mice at 4 weeks after transplanted. (C) Tumor size measured every week after treated with doxorubicin. (D) Tumor weight measured at 4 weeks after transplanted.

Table S1: Base data of patient for survival analysis in GSE21257

	low	high	*P* value
n	31	22	
age	21.24	15.14	0.072
gender			0.2193
male	22	9	
female	12	0	

Table S2. PCR primers information

**Gene**	**Accession Number**	**Description**	**5'-Primer-3'**
NRF2	NM_006164.5	F	AGTCCAGAAGCCAAACTGACAGAAG
		R	GGAGAGGATGCTGCTGAAGGAATC
KEAP1	NM_203500.2	F	ATTCAGCTGAGTGTTACTACCC
		R	CAGCATAGATACAGTTGTGCAG
CUL3	NM_003590.5	F	GACAAATCAACGGAAGAACCAA
		R	TCCTTCTTCAGAAACAAGAGCT
SOD1	NM_000454.5	F	GCGTTTCCTGTCTTTGTACTTT
		R	GTTGCCTGGAACTTTGAGAAG
SOD2	NM_000636.4	F	CGCCCTGGAACCTCACATCAAC
		R	AACGCCTCCTGGTACTTCTCCTC
GPX1	NM_000581.4	F	GTTGCCTGGAACTTTGAGAAG
		R	CTCGATGTCAATGGTCTGGAAG
GPX4	NM_002085.5	F	ATGGTTAACCTGGACAAGTACC
		R	GACGAGCTGAGTGTAGTTTACT
DDRGK1	NM_023935.3	F	GAGTACCTGAAACTGAAGGAGG
		R	TGGACTGCTTGATGTAGTTGAT
